# Retracted: The Anti-Inflammatory Effects of Shinbaro3 Is Mediated by Downregulation of the TLR4 Signalling Pathway in LPS-Stimulated RAW 264.7 Macrophages

**DOI:** 10.1155/2020/7859157

**Published:** 2020-09-29

**Authors:** Mediators of Inflammation

At the request of the authors, the article titled “The Anti-Inflammatory Effects of Shinbaro3 Is Mediated by Downregulation of the TLR4 Signalling Pathway in LPS-Stimulated RAW 264.7 Macrophages” [1] has been retracted. The authors said that while they were conducting further studies on the inflammatory effects of Shinbaro3, they were not able to confirm the results in this article and the Western blots data shown in Figures 3b, 4a, 4b, 6b need further verification and validation; the original uncropped and unadjusted Western blots are unavailable. The conclusions may lack reliability due to inconsistencies in the results and therefore they are repeating the experiments.
